# Equine Facial Action Coding System for determination of pain-related facial responses in videos of horses

**DOI:** 10.1371/journal.pone.0231608

**Published:** 2020-11-03

**Authors:** Maheen Rashid, Alina Silventoinen, Karina Bech Gleerup, Pia Haubro Andersen

**Affiliations:** 1 Dept. Computer Science, University of California Davis, Davis, California, United States of America; 2 Dept. Anatomy, Physiology and Biochemistry, Swedish University of Agricultural Sciences, Uppsala, Sweden; 3 Dept. Clinical Sciences, University of Copenhagen, Taastrup, Denmark; 4 Dept. Clinical Sciences, Swedish University of Agricultural Sciences, Uppsala, Sweden; University of Minnesota, UNITED STATES

## Abstract

During the last decade, a number of pain assessment tools based on facial expressions have been developed for horses. While all tools focus on moveable facial muscles related to the ears, eyes, nostrils, lips, and chin, results are difficult to compare due to differences in the research conditions, descriptions and methodologies. We used a Facial Action Coding System (FACS) modified for horses (EquiFACS) to code and analyse video recordings of acute short-term experimental pain (n = 6) and clinical cases expected to be in pain or without pain (n = 21). Statistical methods for analyses were a frequency based method adapted from human FACS approaches, and a novel method based on co-occurrence of facial actions in time slots of varying lengths. We describe for the first time changes in facial expressions using EquiFACS in video of horses with pain. The *ear rotator* (EAD104), *nostril dilation* (AD38) and lower face behaviours, particularly *chin raiser* (AU17), were found to be important pain indicators. The *inner brow raiser* (AU101) and *eye white increase* (AD1) had less consistent results across experimental and clinical data. Frequency statistics identified AUs, EADs and ADs that corresponded well to anatomical regions and facial expressions identified by previous horse pain research. The co-occurrence based method additionally identified lower face behaviors that were pain specific, but not frequent, and showed better generalization between experimental and clinical data. In particular, *chewing* (AD81) was found to be indicative of pain. Lastly, we identified increased frequency of *half blink* (AU47) as a new indicator of pain in the horses of this study.

## 1 Introduction

Pain is a sign of disease, and early recognition of pain may improve welfare and treatment of otherwise disabling diseases in horses. While self-reporting is the gold standard for assessment of pain in verbal humans [1], there are no measures available for the aversive components of pain in non-verbal mammals, including the horse [2]. The IASP definition of pain states that “the inability to communicate verbally does not negate the possibility that an individual is experiencing pain” [3], referring to adults, neonates, infants, as well as animals unable to communicate. This has brought attention to communication of pain conveyed by non-verbal behaviours, such as bodily behavior, and visible physiological activity such as muscle tremor and facial expressions. During the last decades, a plethora of pain scales based on pain-related bodily behavior has been developed for horses [4–9]. Research in facial expressions as indicators of pain in horses is a more recent contribution [10, 11]. In one pain study [11], pain was induced in otherwise healthy and trained horses using short-term acute pain induction models, whereas horses in another study [10] experienced postoperative pain from castration. Despite many differences in the conditions and methodology, these two very different studies identified and described facial activity in the same regions of the face, corresponding to moveable facial muscles related to the ears, eyes, nostrils, lips, and chin. However, differences were also present. Dalla Costa et al. [12] later identified a classifier that could estimate the pain status of the animal based on the facial activities coded, confirming that the categories used for scoring were related to the pain state of the horse. Due to differences in both experimental approaches and descriptions of the facial activities observed, a detailed comparison of the facial activities during pain in the two mentioned studies has not been done.

In humans, the Facial Action Coding System (FACS) provides a recognized method for identifying and recording facial expressions based on the visible movement of the underlying facial muscles [13]. The coding requires extensive training, and reliable coding can be expected from certified coders. Recently, Wathan et al. [14], on the basis of FACS methodology, developed the Equine Facial Action Coding System (EquiFACS) for horses. EquiFACS exhaustively describes all observable equine facial behavior in three categories: 17 Action Units (AUs), four Ear Action Descriptors (EADs) and seven Action Descriptors (ADs). FACS coding uses detailed frame-by-frame video observation of facial muscle movement, as well as changes in facial morphology (e.g., the position of the eyebrows, size/shape of the mouth, lips, or eyelids, the appearance of various furrows, creases, bulges of the skin) to determine which AU(s) occurred. Inter-observer agreement is good-to-excellent for spontaneously generated facial behavior in more than 90% of the action units in humans [15] scored by trained and certified FACS readers.

The work by Wathan [14] showed that facial movements can be coded reliably only from video sequences and provide precise information about times of onset and offset of the individual AUs. The FACS systems exhaustively code all facial activity observed, not only what is thought to be pain-related. Any interpretations of the emotional meaning of the observed AUs occur post-coding, as the coding system itself is entirely atheoretical.

Pain-related facial responses in horses have never been described using EquiFACS. While the methodology now exists for the coding of horse facial activity, no methods exist for the interpretation of the results. Research on *human* facial expressions of pain is mature and extensive. Kunz et al. [16] presents a systematic review of studies on human facial expressions of pain and describe current approaches for the identification of AUs associated with pain. One approach for defining an AU as pain related is for it to occur frequently, i.e. forming more than 5%, a heuristically set limit, of total AU occurrences in pain state [17]. The second approach is to define an AU as pain related if it occurs more frequently during pain than during baseline [18]. Most often both criteria are applied after each other [19], resulting in a set of AUs that are both frequent and distinct to pain. These methods have never been investigated in horses. Additionally they do not take into consideration the temporal patterns of co-occurring facials actions into consideration, which are increasingly recognized as important for interpretation of facial expressions [20].

Therefore, the aims of this study were to code facial expressions of horses before and during acute experimental pain, and to develop and test statistical approaches that define pain-related facial movements in EquiFACS.

We used videos from a published experiment of acute pain [11] where the horses were habituated to the surroundings and filming conditions, rendering the horses minimally influenced by external input. To explore our models’ ability to generalize to horses in a less controlled environment we also collected and EquiFACS coded videos of horses with and without pain in a clinical setting.

We expected that EquiFACS analysis of painful horses would indicate facial activities in the same anatomical regions as pointed out by the Horse Grimace Scale [10] and the Pain Face [11], and that the statistics based on frequency and the temporal information of the EquiFACS coding could be used to identify facial expressions of pain in videos of horses with experimental and spontaneously occurring pain.

## 2 Horse pain dataset

### 2.1 Experimental pain data

We used videos of six healthy horses of different breeds, five mares and one gelding, aged 3–14 years, recorded during a study of horses subject to acute short-term pain [11]. Briefly, horses were stabled at the research facility for at least ten days before the study, and were positively reinforced during this time to stand in the trial area while wearing only a neck collar. These conditions were designed to increase the horse’s comfort in trial settings, reducing the risk of external factors influencing the horse. Baseline recordings (using Canon Legria HF S21, Canon Inc., Tokyo, Japan) were obtained on the day of the experiment. Acute short term ischemic pain was induced by the application of a pneumatic blood pressure cuff placed on a forelimb and the session was recorded for 20 minutes, while pain behaviour was observed and scored using a modified version of a composite measure pain scale [21].

Video clips of 30 seconds duration were selected from the baseline period, and during nociceptive stimulation, at the first occasion where the profiled horse was within the frame for 30 seconds. This resulted in two videos per horse before and during pain, totaling twelve videos.

### 2.2 Clinical pain data

Twenty-one horses admitted to a horse clinic for either treatment of a disease (n = 11), or control/farriery (n = 10) were filmed with a handheld video camera (Canon Legria, Tokyo, Japan, in HD quality), not restrained and in their observation stall in the premises of The University Animal Hospital Copenhagen or Sweden. Their age ranged from 3 to 17 years (median 8 years) and breeds included warm blood horses (n = 11), trotters (n = 8) and Icelandic horses (n = 2). They were filmed from outside the box with hand held cameras, at the earliest 6 hours after being installed in the box without further acclimatization. Inclusion criteria were owners’ consent for research purposes and exclusion criteria were horses that displayed obvious bodily pain behaviour.

Three veterinarians (two females and one male, with more than 10 years of personal experience with horses) assessed pain level based on their clinical experience as either ‘Severe Pain’, ‘Moderate Pain’, or ‘No Pain’, for each horse without prior knowledge of the horses. To obtain a single pain label, we used majority voting between raters. That is, if at least two of the three raters labeled a video as either ‘Moderate’ or ‘Severe’ pain, the video was labeled as ‘Pain’, else the video was labeled as ‘No Pain’. This resulted in 7 pain and 14 no-pain videos. The video clips were FACS annotated by a single certified EquiFACS coder without prior knowledge of the horses.

### 2.3 Equine facial action coding system

Equine Facial Action Coding System, as described by Wathan et al. [14] was used for a complete annotation of all videos. The system consists of 17 Action Units (AUs), and 11 Action descriptors (ADs), of which four are Ear Action Descriptors (EADs). While AUs represent the contraction of a particular muscle or muscle group, ADs describe a movement caused by either an undetermined muscular basis, or by deep muscles. *For simplicity all EquiFACS codes are referred to as AUs in the following text*.

All films were coded in a blinded manner by a single certified EquiFACS coder without knowledge of the study horses with inter-rater agreement >70% and intra-rater agreement 93%. A complete list of the 28 codes [14] were entered into the annotation software (freeware ELAN [22]). The video clip was first viewed in normal speed. Following, over at least three slow motion, or frame-by-frame, re-runs the annotator coded three regions of the horse face—the ears, upper face, and lower face—and noted the appearance and disappearance of all facial activity. In addition, it was noted if a specific region was out of the frame and therefore not codable.

The resulting dataset contains the occurrence of different AUs, time of their onset, offset, duration, and their temporal overlap with other active AUs. In the statistics section we refer to each period of AU activation—the contraction of muscle or muscle groups associated with the AU—as an AU occurrence. The duration of an AU occurrence is the period of time that elapses between the start and end of its activation. The frequency of an AU in a video sequence is the number of times it is activated during the video for that AU.

## 3 Discovering pain AUs

The EquiFACS datasets derived from experimental and clinical videos was used to identify the action units most useful for the identification of pain in a data-driven manner. AUs associated with head and neck movement were excluded as they do not correspond with facial expressions.

We used a paired t-test for mean values for experimental data, and unpaired t-test for mean values for clinical data to test significance. The number of times an AU occurs within an observation was used for the t-test.

### 3.1 Human FACS Interpretation (HFI) method

As laid out by Kunz et al in a systematic review on human facial expressions of pain [16], we used a two step approach in determining pain AUs. First, AUs that form more than 5% of all AU occurrences in pain videos were selected, meaning that an AU was selected if the number of times it was active in pain videos formed more than 5% of the total number of times any AU was active. From these, the AUs that occurred more frequently in pain than in no-pain videos were determined as the final pain related AUs. To account for unequal number of pain and no-pain videos, AU frequency for pain and no-pain groups was normalized by the number of videos in each group before comparison.

### 3.2 Co-Occurrence method

While the method presented above (Section 4.1) was simple, it does not take into consideration the temporal distribution of onset-offset of the various AUs. AUs that comprise a pain expression are likely to co-occur, i.e. occur together, in a pain state, and are likely to co-occur with a different set of AUs in a no-pain state.

We therefore developed a novel method for describing pain expressions by identifying AUs that occur together in a given period of time. Instead of looking at only frequency and distinctiveness, we compared patterns of co-occurrence of AUs between pain and no-pain states to discover the AUs most indicative of pain.

For comparison of the patterns, we built a graph to capture the co-occurrence relationships between AUs. Each node represented an AU and edges between nodes were weighted by how often they occurred together. We then inspected how edge weights changed between pain and no-pain videos, and selected AUs that exhibited the largest change as pain AUs. All AUs that were active during a pre-defined slice of time—an Observation Window—were counted as co-occurring. This information was available since we recorded the start and end time of each AU activation (see Section 3.3).

More specifically, we built a ‘Co-occurrence Graph’ each for pain—*G*_*P*_—and no-pain—*G*_*NP*_—states. The graph was represented as a *N* × *N* adjacency matrix, where *N* is the total number of annotated action units, and value in row *i* and column *j* of the matrix represents the edge from AU *i* to AU *j*, and is weighted by the fraction of times AU *j* occurs in the same Observation Window as action unit *i*. For example, if AU *j* occurs together with AU *i* in 5 time slices, and AU *i* occurs in 10 time slices in total the value in row *i*, column *j*—referred to as *G*^*i*, *j*^—would be 5/10 = 0.5. The diagonal of this matrix was set to zero. The Co-occurrence Graph is directed, meaning that the value in *G*^*i*, *j*^ need not equal *G*^*j*, *i*^ since AU *i* and *j* can occur in a different total number of Observation Windows.

Using fraction, or relative co-occurrence, rather than raw co-occurrence count, to weigh each edge acts as a normalization procedure such that AUs that occur more frequently (such as blinking) do not have higher edge weights than AUs that occur less frequently. Edge values also become easily interpretable as they capture the co-occurrence rate of any two AUs relative to other co-occurring AUs, and are bounded between 0 and 1.

Following, we subtracted the adjacency matrix of no-pain co-occurrence graph from the adjacency matrix of pain co-occurrence graph to obtain a ‘Difference Graph’, *G*_*D*_.

$$\begin{array}{c} G_{D}=G_{P}-G_{NP} \end{array}$$

*G*_*D*_ captures changes in relative co-occurrence importance between pain and no-pain states. For example a difference value of + 0.3 between AU *i* and *j* implies that AU *j* constitutes 30% more of all co-occurrences in pain than it did in no-pain for AU *i*, and has increased in relative co-occurrence importance. Note that since the pain and no-pain Co-occurrence Graphs are directed graphs, the Difference Graph is also directed.

The AUs with the largest values in the Difference Graph were considered important for pain detection. Given an AU, we calculated its total change in relative co-occurrence importance for all its co-occurring AUs between pain and no-pain states. AUs that showed more than a chosen threshold *t* change were chosen as pain AUs.

More formally, let $G_{D}^{i,j}$ be the value in the *i*th row and *j*th column in the difference graph adjacency matrix. The importance of AU *i* to pain detection, *r*_*i*_, is then calculated by using the following formula that sums column *i*:

$$\begin{array}{c} r_{i}=\sum_{j}^{N}\text{max}\left\{G_{D}^{j,i},\;0\right\} \end{array}$$

In other words, *r*_*i*_ sums the change in relative co-occurrence AUs that co-occur with AU *i* experience. Using column wise summation helps highlight AUs that influence the relative co-occurrence of other AUs. A row wise summation, on the other hand, would disproportionately highlight AUs that only exhibit large changes in relative co-occurrence because they occur once or close to once in the entire dataset. By ignoring decreases, or negative values, in the summation, we avoided AUs that were negatively correlated with pain.

The threshold for selecting pain AUs, *t*, is done using the following formula, where *R* is the set of all *r*_*i*_, *R* = {*r*_*i*_, …, *r*_*n*_}:

$$\begin{array}{c} t=\alpha \;(\text{max}\;R-\text{min}\;R)+\text{min}\;R \end{array}$$

where *α* is a value between 0 and 1. At *α* = 0.5, the threshold is equal to the mid-range of *r*_*i*_ across all AUs.

This selection method thus selects AUs that exhibit a large change in relative co-occurrence between painful and non-painful states. However, the selected AUs need not occur together in the same time slice.

#### 3.2.1 Conjoined pain AUs

For AUs to configure a pain expression they should co-occur in the same Observation Window. They should also occur more frequently in pain rather than no-pain states. We refer to these as conjoined AUs.

This equates to finding a cluster of AUs in the Difference Graph that are all connected to each other, and have positive edge weights. We used a standard method in graph theory—the Bron-Kerbosch algorithm [23]—to find sets of AUs that satisfy these two conditions. We considered any two AUs, *i* and *j* to be connected with a positive edge weight if both $G_{D}^{i,j}$ and $G_{D}^{j,i}$ have a positive value. For every set, we summed its positive edge weights in *G*_*D*_ and selected the set with the highest sum as our final conjoined pain AUs.

### 3.3 Observation Window Size (OWS)

The Observation Window Size determines how close in time two AUs must occur to be considered as co-occurring. For example if two AUs occur within the same 5 second slice, with a *OWS* = 5, they would be counted as co-occurring. With longer OWS, more AUs will probably co-occur, simply because of the continued facial activities of the horse.

Our datasets comprised of 30 second video clips. We used a sliding window based approach to split each video into shorter clips where the step size is set to half the OWS. For example with *OWS* = 5 a 30 second video will be split into 11 shorter clips of duration 5 seconds, starting at times 0, 2.5, 5, 7.5 and so on, seconds. We explored OWS set to 2, 5, 10, 15, 20 and 30 seconds.

By exploring OWS of increasing length, we could capture AU co-occurrence dynamics of varied time length. Each of these shorter clips were treated as separate pain or no-pain observations. A smaller OWS helps increase the size of our dataset so that more reliable assertions can be made.

### 3.4 Predictive values

We inspected the power of specific AUs at reliably predicting pain. If the AU, or set of AUs, are active in a video clip, we marked it as a pain video. Otherwise we marked it as a no pain video. These pain and no-pain predictions were then compared against the ground truth labels to determine the positive and negative predictive value of the AU set.

In addition, we report video level results, where the pain prediction label of the majority observation windows determines the pain prediction label of the entire video.

### 3.5 Pain observation probability

Given a randomly selected video segment of fixed time length, we inspect the likelihood of observing AUs found to be associated with pain (pain AUs). We also inspect how this likelihood differs between the pain and no-pain groups.

Specifically, AUs that are associated to pain by both the HFI and Co-Occurrence methods are selected as the pain AUs. For all time segments in the experimental pain dataset of predefined length—the observation window size (OWS)—we report the percentage of time segments that have a given number of pain AUs activated. In addition to the OWS mentioned above (Section 4.3), we also used an OWS of 0.04 seconds as a proxy for still image based observation since it corresponds to one frame in a 25 frames per second film. We report the likelihood of observing AUs associated with pain in observation windows from pain videos, as well as no-pain videos. Finally, we inspect the percentage difference in these likelihoods between the pain and no-pain groups. For specified OWS, *o*, and, number of pain AUs, *n*, $p_{P}^{n,o}$ and $p_{NP}^{n,o}$ denote the probability of observing *n* pain AUs in a time segment of *o* length in pain videos (*P*) and no-pain (*NP*) videos respectively. The percentage difference was then calculated using the following standard formula:

$$\begin{array}{c} \text{Percentage}\;{\text{Difference}}_{n,t}=\frac{p_{P}^{n,o}-p_{NP}^{n,o}}{\frac{|p_{P}^{n,o}+p_{NP}^{n,o}|}{2}}\times 100 \end{array}$$

## 4 Results

### 4.1 Human FACS Interpretation (HFI)

Table 1 summarizes the AUs that passed the frequency and distinctiveness criterion for selection, along with the percentage of total AU occurrences each comprised, and the percentage difference in frequency each exhibited between experimental pain and no-pain videos.

**Table 1:** AUs found to be associated with pain using the Human FACS interpretation method for experimental data.

Experimental Data Pain AUs with HFI Method								
Action Unit	Chin Raiser (AU17)	Nostril Dilator (AD38)	Half Blink (AU47)	Ear Rotator (EAD104)	Eye White Increase (AD1)	Inner Brow Raiser (AU101)	Blink (AU145)	Ears Forward (EAD101)
Percentage of all Pain video AUs	7.23%	10.54%	12.35%	13.86%	5.72%	13.25%	7.83%	8.73%
More Frequent in Pain Videos	✓	✓	✓	✓	✓	✓	✗	✗
Percentage Difference	90.91%	69.23%	56.25%	42.11%	17.14%	2.30%	-14.29%	-18.75%

*Inner brow raiser* (AU101), *half blink* (AU47), *chin raiser* (AU17), *ear rotator* (EAD104), *eye white increase* (AD1), and *nostril dilator* (AD38) were associated with pain, while, of the 5% most frequent action units, *blink* (AU145) and *ears forward* (EAD101) were not. Of the selected AUs the most pronounced percentage difference in pain and no-pain frequency is for *chin raiser* (AU17) at 90.91%, while *inner brow raiser* (AU101) was barely more frequent in pain videos at just 2.3%.

### 4.2 Co-Occurrence method

Unlike the HFI Method, the Co-Occurrence method for feature selection relies on temporal information to determine pain AUs. For each OWS we determined the relevant AUs and also reported their p-value. Table 2 shows the AUs selected for each observation window size, and for two different threshold values with *α* = 0.5 and 0.3.

**Table 2:** Pain AUs selected by the Co-Occurrence method for experimental data.

Experimental Data Pain AUs with Co-Occurrence Method																
OWS (sec)	*α*	Ear Rotator (EAD104)	Half Blink (AU47)	Nostril Dilator (AD38)	Inner Brow Raiser (AU101)	Chin Raiser (AU17)	Eye White Increase (AD1)	Lip Presser (AU24)	Blink (AU145)	Sharp Lip Puller (AU113)	Ears Forward (EAD101)	Chewing (AD81)	Upper Lid Raiser (AU5)	Nostril Lift (AUH13)	Tongue Show (AD19)	Lip Pucker (AU18)
2	0.5	✓	✓	✓	✓	✓	✓					✓				
	0.3	✓	✓	✓	✓	✓	✓	✓	✓		✓	✓			✓	✓
		(p<0.01)	(p<0.01)	(p<0.001)	(p = 0.720)	(p<0.001)	(p = 0.372)	(p = 0.660)	(p = 0.266)		(p = 0.131)	(p<0.001)			(p<0.05)	(p = 0.258)
5	0.5	✓	✓	✓	✓	✓	✓	✓	✓	✓	✓	✓				
	0.3	✓	✓	✓	✓	✓	✓	✓	✓	✓	✓	✓			✓	✓
		(p = 0.055)	(p<0.01)	(p<0.001)	(p = 1.000)	(p<0.001)	(p = 0.582)	(p = 0.236)	(p = 0.695)	(p = 0.292)	(p = 0.277)	(p<0.01)			(p<0.05)	(p = 0.437)
10	0.5	✓	✓	✓	✓	✓	✓	✓	✓	✓	✓	✓	✓			
	0.3	✓	✓	✓	✓	✓	✓	✓	✓	✓	✓	✓	✓		✓	✓
		(p = 0.123)	(p<0.01)	(p<0.001)	(p = 0.928)	(p<0.01)	(p = 0.712)	(p = 0.363)	(p = 0.517)	(p = 0.344)	(p = 0.225)	(p<0.05)	(p = 1.000)		(p<0.05)	(p = 0.442)
15	0.5	✓	✓	✓	✓	✓	✓	✓	✓	✓	✓	✓	✓			
	0.3	✓	✓	✓	✓	✓	✓	✓	✓	✓	✓	✓	✓		✓	✓
		(p = 0.160)	(p<0.05)	(p<0.01)	(p = 0.740)	(p<0.01)	(p = 0.895)	(p = 0.415)	(p = 0.701)	(p = 0.399)	(p = 0.240)	(p = 0.058)	(p = 0.811)		(p = 0.066)	(p = 0.260)
20	0.5	✓	✓	✓	✓	✓	✓	✓	✓	✓	✓	✓	✓	✓	✓	
	0.3	✓	✓	✓	✓	✓	✓	✓	✓	✓	✓	✓	✓	✓	✓	✓
		(p = 0.270)	(p<0.05)	(p<0.05)	(p = 0.586)	(p<0.05)	(p = 1.000)	(p = 0.581)	(p = 0.339)	(p = 0.504)	(p = 0.342)	(p = 0.104)	(p = 0.662)	(p = 0.266)	(p = 0.107)	(p = 0.389)
30	0.5	✓	✓	✓	✓	✓	✓	✓	✓	✓	✓	✓	✓	✓	✓	✓
	0.3	✓	✓	✓	✓	✓	✓	✓	✓	✓	✓	✓	✓	✓	✓	✓
		(p = 0.175)	(p<0.05)	(p = 0.068)	(p = 0.920)	(p = 0.098)	(p = 0.656)	(p = 0.733)	(p = 0.530)	(p = 0.732)	(p = 0.621)	(p = 0.229)	(p = 0.903)	(p = 0.296)	(p = 0.229)	(p = 0.415)

Values in parenthesis show p-value using paired t-test for mean values. The threshold values are set to include AUs that are above the mid-range value (*α* = 0.5), as well as above the lower third range value (*α* = 0.3), for change in relative co-occurrence.

*Eye white increase* (AD1), *chin raiser* (AU17), *nostril dilator* (AD38), *half blink* (AU47), *inner brow raiser* (AU101), and *ear rotator* (EAD104) are selected across all observation window sizes. All of the selected AUs are selected across multiple observation window sizes.

Of the AUs chosen across all OWS, *half blink* (AU47), *nostril dilator* (AD38), and *chin raiser* (AU17) are statistically significant—i.e. with *p* < 0.05—across almost all OWS. On the other hand, *inner brow raiser* (AU101), and *eye white increase* (AD1) fail to show statistical significance across any observation window size. This is echoed in findings from Section 5.1, where *inner brow raiser* (AU101) is barely more frequent in pain videos compared to no-pain videos, and *eye white increase* (AD1) barely constitutes more than 5% of AU occurrences in pain videos.

Using a smaller observation window size not only accounts for briefer periods of pain expression, but also increases the number of data points for analysis. As a result with *α* = 0.5, ∼71% of AUs selected with an OWS of 2 seconds show statistical significance. In contrast only one, or ∼7%, of selected AUs show statistical significance when using an observation window size of 30 seconds.

*Chewing* (AD81), demonstrates statistical significance, and is chosen as a pain AU across almost all OWS. *Chewing* (AD81) is not a frequent action unit, constituting just 2.11% of AU occurrences in pain videos. However, its inclusion demonstrates that it occurs together with other pain AUs and is therefore important.

At *α* = 0.3, more AUs are selected for each OWS, however, the total set of selected AUs across all OWS remains the same.

### 4.3 Conjoined pain AUs

As described in Section 4.2.1, the conjoined pain AUs occur together in the same time slice, and as a group are more frequent in pain rather than no-pain instances. For brevity, we provide results for *OWS* = 2 seconds. *Nostril dilator* (AD38), *chewing* (AD81), *upper lip raiser* (AU10), *chin raiser* (AU17), and *lip pucker* (AU18) are selected.

### 4.4 Clinical data

We applied the same methods for deriving pain AUs on the clinical data described in Section 3.1. The results using the HFI and Co-Occurrence methods are in Tables 3 and 4 respectively. The threshold for co-occurrence AUs was set to the mid-range (*α* = 0.5), and lower third range (*α* = 0.3) as for experimental data.

**Table 3:** AUs found to be associated with pain using the Human FACS interpretation method for clinical data.

Clinical Data Pain AUs with HFI Method						
Action Unit	Half Blink (AU47)	Inner Brow Raiser (AU101)	Blink (AU145)	Nostril Dilator (AD38)	Ear Rotator (EAD104)	Ears Forward (EAD101)
Percentage of all Pain video AUs	10.89%	19.76%	17.34%	13.71%	10.89%	9.68%
More Frequent in Pain Videos	✓	✓	✓	✓	✗	✗
Percentage Difference	20.41%	15.38%	7.23%	6.06%	-56.95%^-^	-74.51%

**Table 4:** Pain AUs selected by the Co-Occurrence method for clinical data.

Clinical Data Pain AUs with Co-Occurrence Method													
OWS (sec)	*α*	Nostril Dilator (AD38)	Blink (AU145)	Inner Brow Raiser (AU101)	Nostril Lift (AUH13)	Half Blink (AU47)	Ear Rotator (EAD104)	Ears Forward (EAD101)	Chewing (AD81)	Chin Raiser (AU17)	Jaw Thrust (AD29)	Lip Pucker (AU18)	Lip Presser (AU24)
2	0.5	✓	✓	✓	✓								
	0.3	✓	✓	✓	✓		✓	✓	✓				
		(p<0.05)	(p = 0.473)	(p = 0.132)	(p<0.001)		(p<0.001)	(p<0.001)	(p<0.05)				
5	0.5	✓	✓	✓	✓			✓					
	0.3	✓	✓	✓	✓		✓	✓	✓				
		(p = 0.621)	(p = 0.904)	(p = 0.208)	(p<0.01)		(p<0.001)	(p<0.001)	(p = 0.208)				
10	0.5	✓	✓	✓		✓							
	0.3	✓	✓	✓	✓	✓	✓	✓	✓				
		(p = 0.796)	(p = 1.000)	(p = 0.572)	(p = 0.068)	(p = 0.491)	(p<0.01)	(p<0.001)	(p = 0.373)				
15	0.5	✓	✓	✓		✓	✓						
	0.3	✓	✓	✓	✓	✓	✓	✓	✓	✓			
		(p = 0.725)	(p = 0.850)	(p = 0.562)	(p = 0.227)	(p = 0.425)	(p<0.01)	(p<0.001)	(p = 0.680)	(p = 0.131)			
20	0.5	✓	✓	✓	✓	✓	✓	✓					
	0.3	✓	✓	✓	✓	✓	✓	✓	✓	✓	✓	✓	
		(p = 0.835)	(p = 0.853)	(p = 0.763)	(p = 0.467)	(p = 0.463)	(p<0.05)	(p<0.01)	(p = 0.775)	(p = 0.185)	(p = 0.160)	(p<0.05)	
30	0.5	✓	✓	✓	✓	✓	✓	✓					
	0.3	✓	✓	✓	✓	✓	✓	✓	✓	✓	✓	✓	✓
		(p = 0.889)	(p = 0.830)	(p = 0.631)	(p = 0.580)	(p = 0.488)	(p = 0.093)	(p<0.05)	(p = 0.783)	(p = 0.277)	(p = 0.163)	(p<0.05)	(p = 0.486)

Values in parenthesis show p-value using unpaired t-test for mean values. The threshold values are set to include AUs that are above the mid-range value (*α* = 0.5), as well as above the lower third range value (*α* = 0.3), for change in relative co-occurrence.

Conjoined pain AUs for *OWS* = 2 were *jaw thrust* (AD29), *nostril dilator* (AD38), *inner brow raiser* (AU101), and *blink* (AU145).

### 4.5 Specific AUs

As discussed in Section 5.1, *inner brow raiser* (AU101) is only slightly more frequent in experimental pain videos than in no-pain videos, with a percentage difference of 2.3%. For the clinical dataset, *inner brow raiser* (AU101) has a much higher percentage difference of 15.38%.

*Chin raiser* (AU17) and *nostril dilator* (AD38) are selected as AUs indicative of pain by all methods described on experimental data. As a simple test, we use their presence as an indicator of pain and evaluate performance on clinical data.

Table 5 (top) shows the positive predictive value (PPV) and negative predictive value (NPV) for pain prediction for each observation. In addition, we report video level results, where the pain prediction of the of majority observation windows determines the pain prediction of the entire video. In either case, the presence of both AU17 and AD38 has a high positive predictive value for all *OWS* < 20. In particular, observing both AUs within the same 15 second interval has an 80% chance of correctly identifying pain. If the majority of 15 second intervals in a 30 second interval show co-occurrence of both AU17 and AD38, then there is a 100% chance of the observation belonging to a pain episode. On the other hand, the absence of both AU17 and AD38 is also a fairly good indicator of no-pain, particularly for *OWS* > 5. Around 7 out of 10 observations where both AUs are absent correctly correspond with no-pain. However around 3 out of 10 times, a pain observation is incorrectly labeled as no-pain.

**Table 5:** Positive and negative predictive value for different OWS on clinical data.

Results on Clinical Data Per Observation								
	Positive Predictive Value (PPV)				Negative Predictive Value (NPV)			
OWS	AD38	AU17	Either	Both	AD38	AU17	Either	Both
2	38.10%	61.54%	39.11%	85.71%	70.03%	67.92%	71.30%	67.28%
5	35.71%	54.55%	35.97%	77.78%	69.52%	68.90%	70.65%	68.47%
10	33.82%	53.85%	33.33%	83.33%	67.57%	69.57%	66.67%	69.70%
15	32.56%	50.00%	31.25%	80.00%	65.00%	69.81%	60.00%	70.69%
20	32.26%	50.00%	30.30%	66.67%	63.64%	70.59%	55.56%	72.22%
30	31.25%	40.00%	27.78%	66.67%	60.00%	68.75%	33.33%	72.22%
Results on Clinical Data Per Video								
	Positive Predictive Value (PPV)				Negative Predictive Value (NPV)			
OWS	AD38	AU17	Either	Both	AD38	AU17	Either	Both
2	37.50%	-	44.44%	-	69.23%	66.67%	75.00%	66.67%
5	30.77%	100.00%	33.33%	-	62.50%	70.00%	66.67%	66.67%
10	35.71%	50.00%	33.33%	100.00%	71.43%	68.42%	66.67%	70.00%
15	33.33%	50.00%	31.25%	100.00%	66.67%	70.59%	60.00%	73.68%
20	31.25%	40.00%	27.78%	66.67%	60.00%	68.75%	33.33%	72.22%
30	31.25%	40.00%	27.78%	66.67%	60.00%	68.75%	33.33%	72.22%

The criteria for determining pain is the presence of *chin raiser* (AU17), *nostril dilator* (AD38), either, or both. Missing values denoted as “–” indicate no observation with required criteria was present.

### 4.6 Probability of observing pain

We record the percentage of observations of fixed time length where a given number of AUs associated with pain are found (Section 4.5). We use *chin raiser* (AU17), *nostril dilator* (AD38), *half blink* (AU47), *inner brow raiser* (AU101), *eye white increase* (AD1), and *ear rotator* (EAD104) as our pain AUs since they are selected by both the Co-Occurrence, and HFI methods. Results for pain and no-pain videos for experimental data are shown in Table 6.

**Table 6:** Percentage of observation windows from experimental data with specified number of pain AUs present.

Experimental No Pain Videos							
Number of AUs	Observation Window Size (Seconds)						
	0.04	2	5	10	15	20	30
≥1	74.07%	91.95%	98.48%	100.00%	100.00%	100.00%	100.00%
≥2	17.58%	65.52%	84.85%	96.67%	100.00%	100.00%	100.00%
≥3	1.31%	26.44%	62.12%	83.33%	94.44%	91.67%	100.00%
≥4	0.27%	11.49%	30.30%	60.00%	66.67%	75.00%	83.33%
≥5	0.00%	4.02%	15.15%	30.00%	50.00%	58.33%	66.67%
6	0.00%	1.15%	3.03%	10.00%	27.78%	41.67%	50.00%
Experimental Pain Videos							
Number of AUs	Observation Window Size (Seconds)						
	0.04	2	5	10	15	20	30
≥1	81.67%	97.70%	100.00%	100.00%	100.00%	100.00%	100.00%
≥2	31.93%	81.03%	96.97%	100.00%	100.00%	100.00%	100.00%
≥3	6.13%	59.20%	84.85%	96.67%	100.00%	100.00%	100.00%
≥4	0.31%	28.16%	72.73%	90.00%	100.00%	100.00%	100.00%
≥5	0.00%	4.02%	24.24%	60.00%	66.67%	66.67%	66.67%
6	0.00%	1.15%	7.58%	23.33%	44.44%	66.67%	66.67%

**Fig 1:**
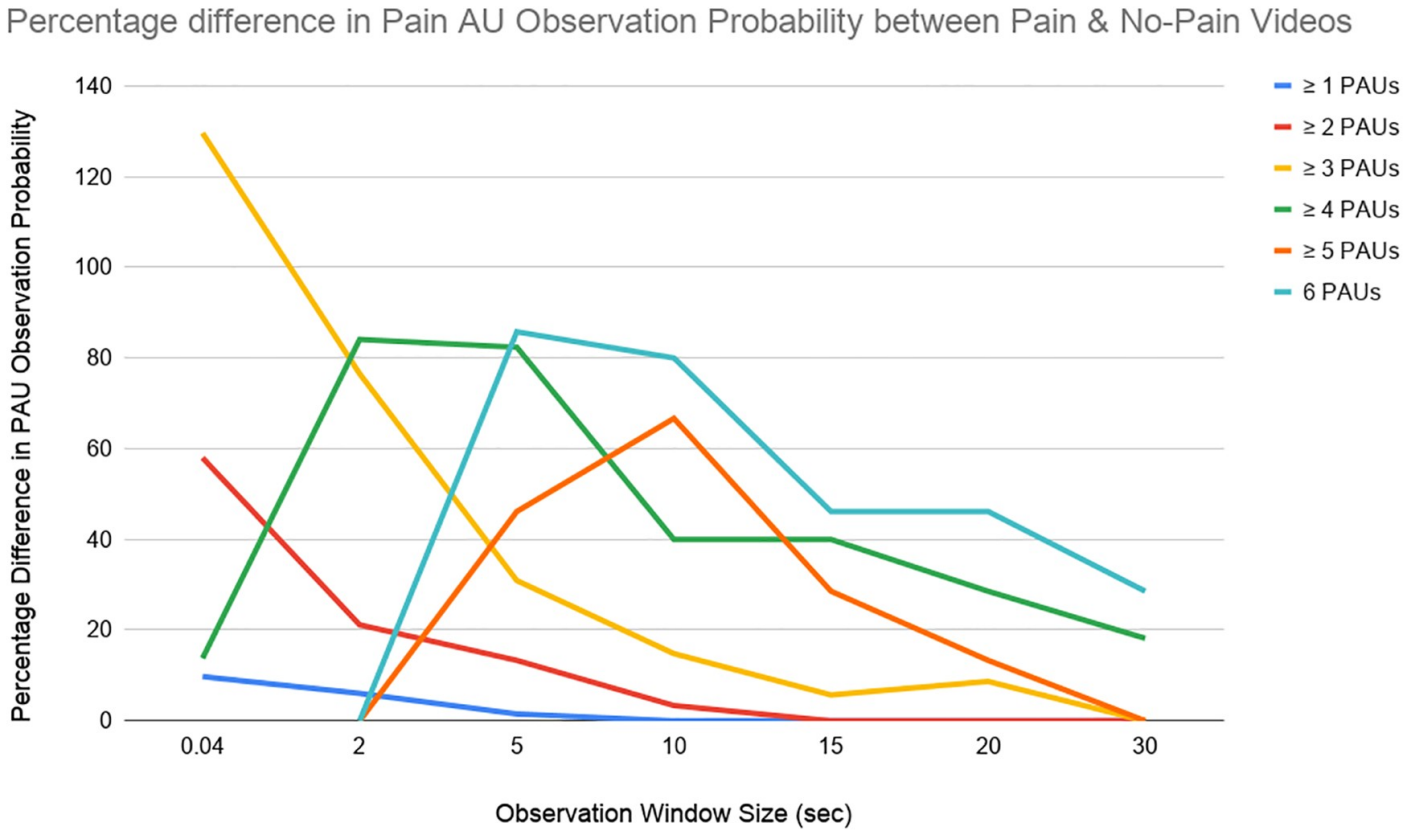
Percentage difference between probability of observing given number of Pain AUs (PAUs) in pain videos (Table 6 *bottom*) from probability of observing given number of PAUs in no pain videos (Table 6 *top*) on experimental data.

Fig 1 shows the percentage difference in probability of observing given number of pain AUs between pain and no-pain videos, i.e. the percentage difference between corresponding cells for pain and no-pain videos in Table 6.

The likelihood of observing at least 3 pain AUs is negligible in still frames (*OWS* = 0.04) at ∼6%, and less than a hundredth chance of observing 4 or more pain AUs. On the other hand, the likelihood of observing at least four pain AUs is much higher for videos, even when observing for 2 seconds at ∼28%.

The likelihood of observing a range of pain AUs is not negligible in no-pain videos. For example, while 60% of 10 second pain clips display 5 or more pain AUs, 30% of no-pain 10 second clips also display 5 or more pain AUs. As observation window size increases, more AUs can be observed together. At the same time, the difference in AU observation probability is reduced between pain and no-pain videos, with a percentage difference of less than 50% across all AU numbers for OWS greater than or equal to 15 seconds.

## 5 Discussion

This study describes for the first time the facial activities in videos of horses in pain by use of the Equine Facial Action Coding System (EquiFACS) [14]. We explored different statistical methods for the analysis of the EquiFACS data.

Using the HFI method on the experimental data, the two most prevalent AUs in painful horse were the *chin raiser* (AU17) and *nostril dilator* (AD38) (Table 1). These two AUs seem to have equivalents in the Horse Grimace Scale [10] as the configurations “mouth strained and pronounced chin” and “strained nostrils and flattening of the profile”; in the Equine Utrecht University Scale for Facial Assessment of Pain (EQUUS-FAP) scale [8] as the configuration (regarding nostrils) “A bit more opened” or “Obviously more opened, nostril flaring” and “Corners mouth/ Lifted a bit” or “Obviously lifted”; and in the Pain Face [11] as the configuration “Edged shape of the muzzle with lips pressed together” and “Nostril dilated in the medio-lateral direction”. This shows that facial expressions of pain as described by EquiFACS occur in the same anatomical regions as described in previous descriptions, such as Pain Face and Horse Grimace Scale.

The third most prevalent AU of the painful horse face was the *half blink* (AU47), which is defined as a reduction of the eye opening by the eyelids, but without complete closure of the eye [14]. The increased rate of half blinks has—to our knowledge—not been documented before as an indication of pain, probably because it is only possible to appreciate this activity from close inspection of video. The action takes place in less than half a second [14]. Decreased eye blink rate has recently been described as a non-invasive measure of stress in horses [24] and the ethogram of the Pain Face contains evidence of increased blinking during pain [11]. The Horse Grimace Scale [10] contains “Orbital Tightening” as a feature with the following description: “The eyelid is partially or completely closed”. The description does not specify the duration of the closure of the eyelid, and may correspond to any of *eye closure* (AU143), *half blink* (AU47), or *blink* (AU145). Since EquiFACS uses temporal information during annotation, the type of eye closure can be determined unambiguously.

EQUUS-FAP scale also focuses on the activity in the eye region, but uses both eye closure and eye widening as indicators of pain [8]. The opening of the eye is described as “obviously more opened eyes”, and increased visibility of the sclera. In EquiFACS these features would be coded as two separate action units, *upper lid raiser* (AU5), and *eye white increase* (AD1), of which AD1 was found by us to be associated to pain. In other studies, increased visibility of eye white has been associated to stress in horses [25].

The “triangular eye” or “worry wrinkles” has empirically been associated to both stress and pain by horse community peoples and veterinarians [24, 25]. In EquiFACS this appearance is coded as the *inner brow raiser* (AU101). Per definition, the activation of this AU increases the perceived size of the eye region, but not the aperture of the eye [14]. This activity also has a parallel in the Horse Grimace Scale where it is described as “tension above the eye area” [10], and in the Equine Pain Face [26] where it is described as “contraction of m. levator anguli oculi medialis”. Given this concurrence we found it remarkable that the frequency of *inner brow raiser* (AU101) was only barely higher in the pain group of this study.

The ears are highly communicative in horses [27]. In this study, increased frequency of *ear rotator* (EAD104) was associated with pain. In the Horse Grimace Scale a “moderately present—stiffly backwards ear” resembles *ear rotator* (EAD104), while the “obviously present stiffly backwards ear” with a wider distance between the tips of the ears resembles the *ear flattener* (EAD103), which has another muscular basis [10]. In the description of the Pain Face, “the lowered ears” with a broader base resembles the *ear rotator* (EAD104), while the “asymmetric ears” described in the Pain Face have no single equivalent in EquiFACS [26]. The EQUUS-FAP scale uses the “backwards ears”; it is not clear if *ear rotator* (EAD104) or *ear flattener* (EAD103) are parallels, or both [8]. It therefore seems important for pain recognition to discriminate between the *ear rotator* (EAD104) and the *ear flattener* (EAD103).

Thus, the EquiFACS and the HFI frequency methods applied from human research point out a number of facial action units that largely correspond well to facial configurations already described in other pain studies. One important exception is the increased frequency of the *half blink* (AU47), which to our knowledge, has not been documented as an action unit with increased frequency during pain. Notably, “the inner brow raiser” (AU101) and the “ears flattener” (EAD103) did not appear as very discriminative of pain.

The HFI method uses each AU frequency independently to determine the subset most correlated with pain. As a result, the selected AUs may not occur at the same time in a pain state. On the other hand, the co-occurrence method captures the relational dynamics of AU occurrences in observation windows of varying time lengths. As a result, the Co-Occurrence method selects AUs that are likely to be observed at the same time during a pain state and therefore shows the appearance of facial expressions of pain. When the Co-occurrence method was used (Table 2) more pain AUs were selected, compared to the HFI method. Generally, AUs of the lower face were selected, specifically *lip pucker* (AU18), *tongue show* (AD19), *lip presser* (AU24), *sharp lip puller* (AU113), and *chewing* (AD81). Regarding nostril movement, *nostril lift* (AUH13) was selected in addition to *nostril dilator* (AD38). Additionally, *eye white increase* (AD1), and *inner brow raiser* (AU101), were selected across all observation time lengths, but were not statistically significant.

While the co-occurrence method identifies AUs that demonstrate a different relational dynamic between pain and no-pain states, the “Conjoined Pain AUs” explicitly identify clusters of AUs that occur together and more frequently in pain than in the no pain states. The method did select both the AUs that demonstrated the strongest association to pain using the HFI and Co-Occurrence methods—*nostril dilator* (AD38), and *chin raiser* (AU17), but also selected AUs associated with lower face movement—*lip pucker* (AU18), *chewing* (AD81)—and nostril movement—*upper lip raiser* (AU10). This may indicate that lower face movements convey indicators of pain that should be further studied.

Not surprisingly, the likelihood of observing multiple pain AUs was strongly linked to the length of observation time. In still images, or OWS of 0.04 seconds, the likelihood of observing more than three pain AUs was negligible at less than half a percent for pain videos, and with little percentage difference from the likelihood of observing the same number of AUs in no-pain videos. In contrast to this, in our limited dataset, observing 4 or more pain AUs in a 5 second observation window was both likely (occurring in 72% of 5 second pain clips), and significantly more likely in a pain video than a no-pain video (percentage difference of 84%). An implication of this may be that observation of video for pain assessment in horses may be of higher value than randomly selected images.

While the experimental dataset was collected under controlled circumstances, with the pain induction providing a kind of gold standard for the occurrence of pain, no gold standard exists for spontaneous pain. The facial expressions of pain are believed to be universal for all species, across different types of pain [18]. It was therefore of interest to investigate how the models developed from experimental data could predict what clinicians consider to be pain.

For the clinical data set, we deliberately did not infer anything about the diagnoses of horses, since even horses that come for control or routine farriery, may be in pain, and some horses may have diseases that are actually not painful. The true pain status of the horses could not be known, and we can therefore only show how a global pain assessment of clinical cases relates to statistical models built on EquiFACS of experimental horses.

The pain AUs selected by the HFI method were not entirely similar between the clinical and experimental data. While *half blink* (AU47), *nostril dilator* (AD38), and *inner brow raiser* (AU101) were selected as in the experimental data, the AUs *ear rotator* (EAD104), *chin raiser* (AU17), and *eye white increase* (AD1) were not selected. On the other hand, *blink* (AU145), was selected in the clinical data, but was not in the experimental data.

The co-occurrence method selected less AUs in clinical data compared to the experimental data when the threshold for AU selection was similar to the experimental situation. Lowering the selection threshold resulted in a similar set of AUs being selected compared to the experimental data with some exceptions; *Eye white increase* (AD1), *upper lid raiser* (AU5), *sharp lip puller* (AU113), and *tongue show* (AD19) were not selected with clinical data, but were selected with the experimental dataset. On the other hand, *jaw thrust* (AD29) was selected with clinical data, but was not selected with the experimental pain dataset.

Similar to the 5% threshold used in the HFI method, the threshold value *α* used in the co-occurrence method is set heuristically, and may lead to different results across different datasets. Its value corresponds to the amount of difference AUs must display in co-occurrence patterns between pain and no-pain states to be selected as pain AUs. Developing a criteria for selecting an optimum selection threshold is an important and interesting direction of future research.

Interesting differences appeared between the clinical and experimental data. AUs corresponding to eye aperture increase (AD1 and AU5) were considered indicative of pain in the experimental dataset, but not in the clinical dataset. Lower face AUs also differed. While experimental data featured *sharp lip puller* (AU113), and *tongue show* (AD19), the clinical data did not and instead featured the *jaw thrust* (AD29). In general, apart from *chewing* (AD81), lower face movements were selected across fewer observation window sizes for clinical data than upper face and nostril movements. We can only speculate about the reasons for these discrepancies, which could be due to differences in the pain experience, pain type (nociceptive acute pain versus chronic or inflammatory pain), pain duration, or reliability of pain/no-pain labels between experimental and clinical data.

The co-occurrence method generally showed overall higher agreement between pain AUs across both datasets than the HFI method. This points to the advantage of co-occurrence over the simple frequency based HFI method. Since the HFI method ignores the temporal dynamics between AUs the method is less able to select discriminative AUs that occur less frequently such as *chin raiser* (AU17). The lack of a gold standard for clinical pain continues to be an unsolved issue. With data that has imperfect labels, the difference between pain and no-pain frequency patterns may be reduced, leading to less consistent results.

To test the pain predictive ability of AUs derived from experimental data in the clinical setting, we used the two AUs most consistently chosen as indicative for pain in the experimental data. The positive predictive values of *nostril dilator* (AU38) and *chin raiser* (AU17) were 100% if these actions were both observed within an Observation Window Size of 10 to 15 seconds. The absence of these actions had a poor negative predictive value, meaning that other actions should be looked for if a horse should be claimed without pain. These observations should be explored further using EquiFACS to increase sensitivity and specificity of pain assessment scales.

One limitation of this pilot study is the low number of experimental horses that the models were built on. While the acclimatization of horses in the experimental setting was an advantage for obtaining as little interference from external inputs as possible, it might at the same time limit generalisation to data with external interference, where there is no gold standard for assessment of pain. We based the presumption of pain on clinically experienced observers’ evaluation, and not the reason for admittance, as the true pain status of these horses can not be known. We used a simple dichotomous pain/no-pain model for this study due to the low number of horses, the lack of a validated pain scale with intensity scoring for video, and the lack of intensity codes in EquiFACS. We could have used both a larger number of experienced clinicians and a larger number of clinical and experimental cases, issues that needed to be balanced against the very resource demanding process of FACS annotation. Finally, this study only investigated the facial activities produced by a single pain modality from experimental data. Clinical data showed more diversity of AUs, which may be due to difficulties with correct pain classification or the co-existence other emotional states. Pain expressions should therefore be studied in a larger number of more diverse horses, during different clinical conditions and with different types of pain.

In conclusion, we have for the first time described the facial activities of one “prototypical” pain face of acute pain in the horse using a Facial Action Coding System. We identified increased frequency of *half blink* (AU47) as an indicator of pain in the horses of this study. The *ear rotator* (EAD104), *nostril dilator* (AD38) and lower face behaviours, particularly *chin raiser* (AU17), were found to be important pain indicators. The *inner brow raiser* (AU101), and *eye white increase* (AD1) had less consistent results across experimental and clinical data. Frequency statistics identified AUs, EADs and ADs that corresponded well to anatomical regions and facial expressions identified by previous horse pain research. Novel co-occurrence based method additionally identified facial behaviors that were pain specific, but not frequent, and showed better generalization between experimental and clinical data. In particular, *chewing* (AD81) was found to be indicative of pain. However, the reported methodologies need further testing in larger sample sizes.

## Supporting information

S1 File Experimental pain data horse 1-12 videos. (ZIP)

S1 Data Experimental pain data FACS, Pain, and horse ID annotation for 1-12 videos. (XLSX)

S2 Data Clinical pain data FACS, and pain annotation for 21 clinical videos. (XLSX)

## References

[1] Hadjistavropoulos T, Craig KD. A theoretical framework for understanding self-report and observational measures of pain: a communications model. Behaviour research and therapy. 2002;40(5):551–570. 10.1016/S0005-7967(01)00072-9 12038648

[2] Flecknell P, Leach M, Bateson M. Affective state and quality of life in mice. Pain. 2011;152(5):963–964. 10.1016/j.pain.2011.01.030 21292396

[3] IASP Taxonomy: International Association for the Study of Pain; 2016;. http://www.iasp-pain.org/Education/Content.aspx?ItemNumber=1698.

[4] Raekallio M, Taylor PM, Bloomfield M. A comparison of methods for evaluation of pain and distress after orthopaedic surgery in horses. Journal of Veterinary Anaesthesia. 1997;24(2):17–20. 10.1111/j.1467-2995.1997.tb00150.x

[5] Price J, Catriona S, Welsh EM, Waran NK. Preliminary evaluation of a behaviour–based system for assessment of post–operative pain in horses following arthroscopic surgery. Veterinary anaesthesia and analgesia. 2003;30(3):124–137. 10.1046/j.1467-2995.2003.00139.x 14498844

[6] Sellon DC, Roberts MC, Blikslager AT, Ulibarri C, Papich MG. Effects of continuous rate intravenous infusion of butorphanol on physiologic and outcome variables in horses after celiotomy. Journal of Veterinary Internal Medicine. 2004;18(4):555–563. 10.1111/j.1939-1676.2004.tb02585.x 15320598

[7] Graubner C, Gerber V, Doherr M, Spadavecchia C. Clinical application and reliability of a post abdominal surgery pain assessment scale (PASPAS) in horses. The Veterinary Journal. 2011;188(2):178–183. 10.1016/j.tvjl.2010.04.029 20627635

[8] van Loon JP, Van Dierendonck MC. Monitoring acute equine visceral pain with the Equine Utrecht University Scale for Composite Pain Assessment (EQUUS-COMPASS) and the Equine Utrecht University Scale for Facial Assessment of Pain (EQUUS-FAP): A scale-construction study. The Veterinary Journal. 2015;206(3):356–364. 10.1016/j.tvjl.2015.08.023 26526526

[9] Gleerup K, Lindegaard C. Recognition and quantification of pain in horses: A tutorial review. Equine Veterinary Education. 2016;28(1):47–57. 10.1111/eve.12383

[10] Dalla Costa E, Minero M, Lebelt D, Stucke D, Canali E, Leach MC. Development of the Horse Grimace Scale (HGS) as a pain assessment tool in horses undergoing routine castration. PLoS one. 2014;9(3):e92281 10.1371/journal.pone.0092281 24647606 PMC3960217

[11] Gleerup KB, Forkman B, Lindegaard C, Andersen PH. An equine pain face. Veterinary anesthesia and analgesia. 2015;42(1):103–114. 10.1111/vaa.12212 PMC4312484 25082060

[12] Dalla Costa E, Pascuzzo R, Leach MC, Dai F, Lebelt D, Vantini S, et al Can grimace scales estimate the pain status in horses and mice? A statistical approach to identify a classifier. PloS one. 2018;13(8):e0200339 10.1371/journal.pone.0200339 30067759 PMC6070187

[13] Ekman P, Friesen WV, Hager JC. Facial Action Coding System. Manual and Investigator’s Guide. 2002;.

[14] Wathan J, Burrows AM, Waller BM, McComb K. EquiFACS: The Equine Facial Action Coding System. PloS one. 2015;10(8):e0131738 10.1371/journal.pone.0131738 26244573 PMC4526551

[15] Sayette MA, Cohn JF, Wertz JM, Perrott MA, Parrott DJ. A psychometric evaluation of the facial action coding system for assessing spontaneous expression. Journal of Nonverbal Behavior. 2001;25(3):167–185. 10.1023/A:1010671109788

[16] Kunz M, Meixner D, Lautenbacher S. Facial muscle movements encoding pain—a systematic review. Pain. 2019;160(3):535–549. 10.1097/j.pain.0000000000001424 30335682

[17] Hampton AJ, Hadjistavropoulos T, Gagnon MM, Williams J, Clark D. The effects of emotion regulation strategies on the pain experience: a structured laboratory investigation. Pain. 2015;156(5):868–879. 10.1097/j.pain.0000000000000126 25734999

[18] Prkachin KM. The consistency of facial expressions of pain: a comparison across modalities. Pain. 1992;51(3):297–306. 10.1016/0304-3959(92)90213-U 1491857

[19] Karmann AJ, Maihöfner C, Lautenbacher S, Sperling W, Kornhuber J, Kunz M. The role of prefrontal inhibition in regulating facial expressions of pain: a repetitive transcranial magnetic stimulation study. The Journal of Pain. 2016;17(3):383–391. 10.1016/j.jpain.2015.12.002 26705973

[20] Krumhuber EG, Kappas A, Manstead AS. Effects of dynamic aspects of facial expressions: A review. Emotion Review. 2013;5(1):41–46. 10.1177/1754073912451349

[21] Lindegaard C, Thomsen MH, Larsen S, Andersen PH. Analgesic efficacy of intra-articular morphine in experimentally induced radiocarpal synovitis in horses. Veterinary anaesthesia and analgesia. 2010;37(2):171–185. 10.1111/j.1467-2995.2009.00521.x 20230568

[22] ELAN. Max Planck Institute for Psycholinguistics, The Language Archive, Nijmegen, The Netherlands;.

[23] Bron C, Kerbosch J. Algorithm 457: finding all cliques of an undirected graph. Communications of the ACM. 1973;16(9):575–577. 10.1145/362342.362367

[24] Merkies K, Ready C, Farkas L, Hodder A. Eye Blink Rates and Eyelid Twitches as a Non-Invasive Measure of Stress in the Domestic Horse. Animals. 2019;9(8):562 10.3390/ani9080562 PMC6721043 31443315

[25] Hintze S, Smith S, Patt A, Bachmann I, Würbel H. Are eyes a mirror of the soul? What eye wrinkles reveal about a horse’s emotional state. PloS one. 2016;11(10):e0164017 10.1371/journal.pone.0164017 27732647 PMC5061373

[26] Gleerup KB, Andersen PH, Munksgaard L, Forkman B. Pain evaluation in dairy cattle. Applied Animal Behaviour Science. 2015;171:25–32. 10.1016/j.applanim.2015.08.023

[27] Wathan J, McComb K. The eyes and ears are visual indicators of attention in domestic horses. Current Biology. 2014;24(15):R677–R679. 10.1016/j.cub.2014.06.023 25093554 PMC4123162

